# Towards a comprehensive characterization of durum wheat landraces in Moroccan traditional agrosystems: analysing genetic diversity in the light of geography, farmers’ taxonomy and tetraploid wheat domestication history

**DOI:** 10.1186/s12862-014-0264-2

**Published:** 2014-12-21

**Authors:** Ali Sahri, Lamyae Chentoufi, Mustapha Arbaoui, Morgane Ardisson, Loubna Belqadi, Ahmed Birouk, Pierre Roumet, Marie-Hélène Muller

**Affiliations:** Département de Production, Protection et Biotechnologies Végétales, Institut Agronomique et Vétérinaire Hassan II, B.P. 6202, Rabat-Instituts, Rabat, Morocco; INRA, UMR 1334, Amélioration Génétique et Adaptation des Plantes méditerranéennes et tropicales (AGAP), 2 place Pierre Viala, F-34060 Montpellier Cedex 1, France

**Keywords:** Genetic diversity, Traditional agrosystems, Variety names, Landraces, Durum wheat

## Abstract

**Background:**

Crop diversity managed by smallholder farmers in traditional agrosystems is the outcome of historical and current processes interacting at various spatial scales, and influenced by factors such as farming practices and environmental pressures. Only recently have studies started to consider the complexity of these processes instead of simply describing diversity for breeding purposes. A first step in that aim is to add multiple references to the collection of genetic data, including the farmers’ varietal taxonomy and practices and the historical background of the crop.

**Results:**

On the basis of interview data collected in a previous study, we sampled 166 populations of durum wheat varieties in two traditional Moroccan agrosystems, in the Pre-Rif and Atlas Mountains regions. Using a common garden experiment, we detected a high phenotypic variability on traits indicative of taxonomical position and breeding status, namely spike shape and plant height. Populations often combined modern (short) with traditional-like (tall) statures, and classical durum squared spike shape (5 flowers/spikelet) with flat spike shape (3 flowers/ spikelet) representative of primitive domesticated tetraploid wheat (ssp. *dicoccum*). By contrast, the genetic diversity assessed using 14 microsatellite markers was relatively limited. When compared to the genetic diversity found in a large collection of tetraploid wheat, it corresponded to free-threshing tetraploid wheat. Within Morocco, the two studied regions differed for both genetic diversity and variety names. Within regions, neither geography nor variety names nor even breeding status constituted strong barriers to gene exchange despite a few significant patterns.

**Conclusions:**

This first assessment of morphological and genetic diversity allowed pointing out some important factors that may have influenced the structure and evolutionary dynamics of durum wheat in Morocco: the significance of variety names, the occurrence of mixtures within populations, the relative strength of seed exchange between farmers and local adaptation, as well as the fate of modern varieties once they have been introduced. Further, multidisciplinary studies at different spatial scales are needed to better understand these complex agrosystems of invaluable importance for food security.

**Electronic supplementary material:**

The online version of this article (doi:10.1186/s12862-014-0264-2) contains supplementary material, which is available to authorized users.

## Background

With the Green Revolution, most of breeding effort has been devoted to the development of elite cultivars adapted to favorable lands where high input agriculture is predominantly practiced [1]. The attention dedicated to marginal lands is more recent [2,3]. These areas, characterized by specific and heterogeneous environmental conditions, reduced economic return and/or severe limitations for intensive agricultural use, host a large share (if not the majority in Africa, Asia, Central and South America) of rural populations. There, smallholder farmers practice low-input traditional agriculture and grow a large diversity of crop species [4]. One of the key production factors ensuring the sustainability of these agricultural systems is the set of traditional varieties (landraces) having evolved in these places for generations under a combination of environmental and human pressures [4]. Their high genetic diversity buffers against spatial and temporal heterogeneity and enhances resilience to abiotic and biotic stresses compared to modern varieties [5,6]. These landraces have often been considered as resources for contemporary agriculture; they have been used in breeding programs to enlarge the genetic diversity of modern genetic pools and to improve the adaptation of modern cultivars to abiotic or biotic constraints for instance, [7-9]. Considering the incapacity of modern breeding to provide adapted varieties to traditional agrosystems [10], landraces need to be considered for themselves [11], as the basis for food security. Efforts have to be devoted to their conservation and their use, especially in their place of origin [12].

Studies of landraces often focus on describing their genetic diversity in order to identify valuable and diversified genetic resources; the genetic information is generally only coupled with the geographical origin of the accessions [13,14]. To implement coherent actions for the conservation and use of the diversity present in traditional agrosystems, it is necessary to bridge the gap between the diversity observed within and across fields and the historical and current processes that have been shaping such diversity. These processes involve the interaction of multiple factors: Human practices such as farming practices, seed regeneration for the upcoming agricultural season, seed storage conditions, consumption habits, as well as seed exchanges within and between communities of farmers, generate selection pressures, drift or founder effects [15-17]. Particularly, cultural barriers act jointly with geographical ones on reproductive isolation, limiting or extending gene flow within a region [18,19]. In parallel, environmental conditions such as climate and soil characteristics are likely to strongly affect the evolutionary dynamics of landraces through heterogeneous selection pressures [20,21]. Moreover, extreme climatic events can lead to local extinction and seed renewing affecting genetic diversity patterns [22]. Finally, the recent introduction of modern varieties might break the existing equilibrium, but can at the same time enrich the local genetic diversity [23].

Disentangling these mechanisms requires as first step integrating additional information to the morphologic and genetic characterization of landraces, in order to consider them in their environmental and social context [24]. For instance, various studies relying on interviews with farmers have been conducted, such interviews appearing essential to properly interpret the genetic diversity [11,25-27]. Together with the characteristics of the farm and the farmers’ practices, the name of the variety especially appears as a crucial piece of information, since it is most often consciously used by farmers for management, selection, exchanges and uses [28].

Durum wheat (*Triticum turgidum ssp. durum*) is a selfing tetraploid cereal. The outcrossing rate is generally considered lower than 5% by breeders, and has been estimated between 1 and 3% in Ethiopian landraces [29]. Durum wheat history began with the domestication of wild emmer (*Triticum turgidum* ssp. *dicoccoides)* in the mountains of Fertile Crescent around the 8th millennium BC. Firstly it led to a non-brittle hulled subspecies (*T. t.* ssp. *dicoccum*) which was one of the first cereals domesticated [30]*.* Then, human selection of mutations at multiple loci in particular at *Tg* – tenacious glume – and *Q* loci, [31-33] gave rise to traits such as square-shaped spike, soft glumes and non-hulled grains improving threshing efficiency [34,35] and facilitating widespread cultivation. This phenotypic evolution together with hybridization between different forms [36] led to free-threshing subspecies (*T. t.* ssp. *polonicum, T. t.* ssp. *carthlicum, T. t.* ssp. *turgidum, T. t.* ssp. *durum*). Hulled and free-threshing forms (from 7th and 3rd millenium BP respectively) played a crucial role in the development of Mediterranean civilizations. They were widely spread out with the early agricultural movements leading to agriculture systems based on tetraploid wheat. Nowadays, elite durum varieties and landraces grown in different environments are of major importance for grain production in the Mediterranean basin [37]. Molecular markers-based studies showed that durum wheat history was associated with a decrease of the level of genetic diversity, from the wild ancestor to the most recent modern varieties [38,39], and that the different free-threshing subspecies, differing mainly in spike traits, were generally not distinguishable genetically [40,41].

In Morocco, tetraploid wheat represents a major crop (20% of cereals surface, www.agriculture.gov.ma) and an important source of staple food. Most of the cultivated varieties in the Great Plains result from breeding programs developed by the Moroccan National Institute of Agricultural Research (INRA) or by international research centres (ICARDA, CIMMYT). These modern varieties are single-genotype based, early flowering spring (i.e. they do not require low temperature to induce flowering and/or they are insensitive to day length) with a short stature due to dwarfing genes, [42] to avoid lodging in relation with nitrogen fertilization. In contrast, in mountainous regions of Morocco (Rif, Central and High Atlas), the traditional agriculture system remains largely based on traditional durum wheat varieties, recognizable with their tall stature allowing the use of straw and grain. In these marginal areas, a diversity of tetraploid wheat types have been reported historically, including hulled wheat (denoted as subsp. *maroccanum* [43] or more classically as ssp. *dicoccum* [44,45]).

A few studies have described the genetic diversity of Moroccan landraces in terms of allele number and specific alleles [46,47]. Kehel et al. [46] detected a structure of the genetic diversity according to geography, water and temperature regimes; noticeably, landraces from the Rif and from the high continental plateaus from eastern High Atlas (including Saharan slope) were differentiated genetically. But in these studies, no other information than geographical location and large scale agro-ecological conditions was incorporated. Chentoufi et al. [48] recently conducted interviews with farmers from the Pre-Rif and the oases of the Atlas Mountains. They inventoried the varietal names and described their geographical distribution. Among their key results, they evidenced that the seed was in majority produced on farm, and that exchange practices included a combination on (mainly) purchase from local markets, supply from friends and relatives, and rarely, supply of modern varieties from agricultural extension offices. In these regions, modern varieties have indeed been introduced at variable levels and Chentoufi et al. [48] showed that they were integrated into the traditional practices of seed exchange and management. Nevertheless, farmers clearly distinguished traditional from modern varieties, and appreciated the traditional varieties especially for the straw and the quality for food processing.

In the present work, we enriched these interviews with genetic and morphologic data to address three questions: (i) How can we locate the Moroccan cultivated populations in the historical background of tetraploid wheat domestication? Do we find the different evolutionary steps described above (subspecies of *Triticum turgidum* and products of modern breeding)? (ii) How is the Moroccan genetic and morphologic diversity structured relative to two factors: the geographic location at small and large scales, and the farmers’ taxonomy (i.e. the varietal names attributed to the cultivated populations). These two factors are potentially important determinants of gene flow through seed exchange or intercrossing. (iii) What are the consequences of the introduction of modern varieties? Do the traditional and modern genetic pools remain distinct and how can the diversity of traditional varieties be affected on the long term?

To address these questions, we used two data sets. One, denoted as the Moroccan sample, is made of a sample of Moroccan farmers’ population, identified by their varietal name and their farmer of origin [48]; the other, denoted as the INRA collection, is a worldwide collection of *Triticum turgidum* conserved at INRA (French National Institute for Agricultural Research) and including wild and domesticated subspecies. We considered three kinds of markers: The spike shape is an indicator of the taxonomical position, the stature of the plants is an indicator of breeding status, and a set of microsatellite markers gives us access to the neutral genetic diversity. The results are presented with respect with these three kinds of markers, and considered together to give insights into our three axes of investigation. For the interpretation, we also made use of the qualitative information gathered by the interviews of Chentoufi et al. [48].

## Results

**Figure 1:**
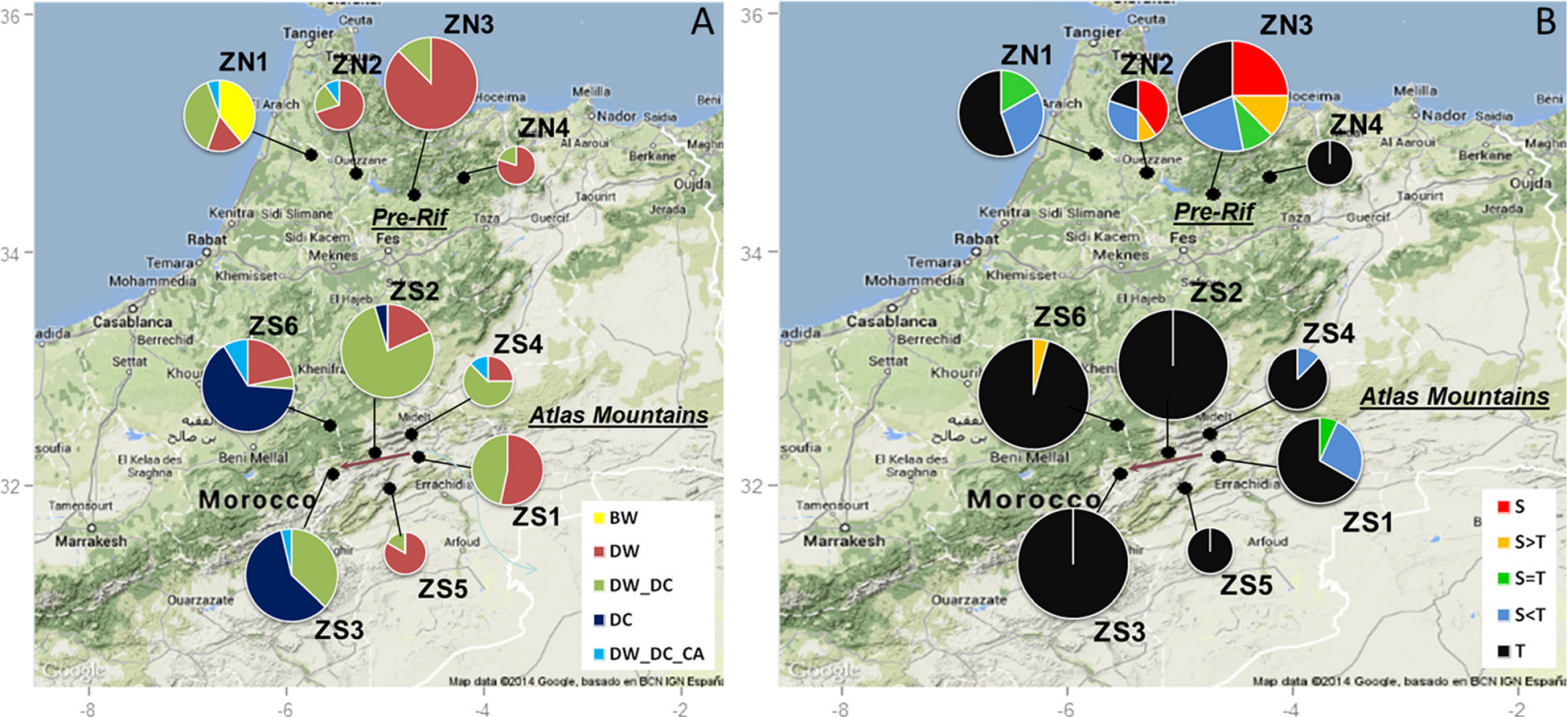
**Maps of the study areas showing the morphological composition of the sampling zones. A**. Frequencies of the different spike-type classes of populations. Each colour represents a spike-type class. BW: populations containing bread wheat. DW (resp. DC): pure populations made of DW (resp. DC) spike types. DW_DC: mixed populations combining DW and DC spike types. DW_DC_CA: mixed populations including DW, DC and CA spike types. **B**. Frequencies of the different stature classes of populations. Each colour represents a stature-class. S. (resp T.): pure populations made of short (resp. tall) statures. S > T, S = T, S < T: mixed populations containing respectively more than 66%, between 33 and 66%, and less than 33% of short statures. Chart pie diameters are proportional to the number of populations sampled in each zone. The arrow in the Atlas Mountains underlines the position of the Ziz valley, from low (ZS1) to high elevation (ZS3) (see text). The map backgrounds have been extracted from Google Maps.

We sampled 166 durum wheat populations in two Moroccan regions (Moroccan sample). These populations belonged to 30 different varieties that were classified as modern or traditional. Each region was subdivided into zones including geographically close villages (Table 1, Figure 1)

**Table 1:** **Summary of the sampling design in Morocco**

**Region**	**Zone**	**Altitude range (m)**	Number of different varieties collected *	Number of sampled populations **
**Pre-Rif**	**ZN1**	187-428	8 (1)	18 (1)
	**ZN2**	191-422	5 (1)	10 (5)
	**ZN3**	180-650	12 (3)	32 (8)
	**ZN4**	872	2	5
	**total**	180-872	18 (3)	65 (14)
**Atlas Mountains**	**ZS1**	1385-1464	5 (1)	15 (1)
	**ZS2**	1593-2133	6	22
	**ZS3**	2160-2387	5	27
	**ZS4**	1662-1674	4	8
	**ZS5**	1293-1296	3	6
	**ZS6**	1492-1894	2	23
	**total**	1293-2387	12 (1)	101 (1)

Altitude was recorded at the village and not at the field level.

* (resp. **) In brackets, number of modern varieties (resp. number of sampled populations belonging to modern varieties).

.

### Morphological diversity in the Moroccan sample

We characterized the height and spike shape of 30 maternal families (denoted as progenies) per population and of two Moroccan modern varieties used as checks.

#### Spike shape variability: geographic structuration and relative inconsistency of its distribution across varietal names

Using a combination of spike shape and flow cytometric analysis, each progeny was assigned to one of four spike types: one corresponding to hexaploid bread wheat (*Triticum aestivum*, BW), and three corresponding to a shape indicative of different subspecies of the tetraploid species *Triticum turgidum* (CA for ssp. *carthlicum*, DC for ssp. *dicoccum* and DW for ssp. *durum*). Even if this description is referring to different subspecies of tetraploid wheat, it cannot be considered sufficient to confidently assign a plant to a subspecies. Variability for spike type was observed among and within populations. Based on within-populations frequencies of spike types, different categories of pure and mixed populations were defined (see Methods for details).

Bread wheat was detected in 7 populations, all located in zone ZN1 of the Pre-Rif (Table 2, Figure 1A), constituting in two cases pure bread wheat populations.

**Table 2:** **Frequencies of spike types and of short progenies within each zone**

**Regions**	**Zones**	**Number of progenies**	**Spike types**				**Short progenies (S)**
			***T. turgidum***			***T. aestivum***	
			**DW**	**DC**	**CA**	**BW**	
**Pre-Rif**	ZN1	402	0.53	0.27	0.06	0.14	0.08
	ZN2	299	0.89	0.03	0.08	0	0.53
	ZN3	959	0.95	0.04	0.01	0	0.38
	ZN4	149	0.9	0.1	0	0	0
**Atlas Mountains**	ZS1	450	0.81	0.19	0	0	0.07
	ZS2	660	0.64	0.36	0	0	0
	ZS3	810	0.11	0.87	0.02	0	0
	ZS4	240	0.84	0.14	0.02	0	0.11
	ZS5	180	0.98	0.02	0	0	0
	ZS6	690	0.25	0.72	0.03	0	0.03

Frequencies have been computed among the progenies sampled in the field and sowed in the common garden.

Within *Triticum turgidum*, two spike types were predominant overall, DC and DW. In the Pre-Rif, ZN1 was characterized by a large diversity of spike types and a notable frequency of mixed populations (DW-DC and DW-DC-CA). The other zones were dominated by the classical DW type (Table 2, Figure 1A). The DC and CA spike-types were always rare at the within-population level.

In the Atlas Mountains, spikes were mainly of DW and DC types. Two groups of zones appeared: ZS1, ZS2, ZS4 and ZS5 were characterized by a high proportion of the DW type. ZS3 and ZS6 were dominated by the DC type with similar overall frequencies, but with a prevalence of pure populations in ZS6. A gradient was detected along the Ziz valley with an increase of DC type frequencies from ZS1 to ZS3. Mixed populations prevailed in the intermediate zone ZS2 by comparison with zones ZS1 and ZS3 where pure populations of respectively DW and DC spike-types were more frequent (Table 2, Figure 1A).

Most of the varieties were represented by several populations. We thus attempted to categorize the varieties according to the morphological composition of these populations (Tables 3 and 4). Some varieties were associated to a given spike type: for instance, modern varieties were of the DW-type, as well as most traditional varieties of the Pre-Rif (e.g. Guemh, Krifla kehla, etc). In the Atlas Mountains, traditional varieties split up in two groups: DC varieties, including pure populations and mixed ones with a majority of DC spike-types, and DW varieties, also including pure populations and mixtures. But these categories reflected tendencies rather than strict correspondences: some varieties assigned to a given category also included outlier populations: For instance, one population referred as Ifermourgh, a DC variety, contained only DW spike-type plants.

**Table 3:** **Occurrence and morphological characteristics of the varieties cultivated in the Pre-Rif region**

**Variety**	**Status**	**Sample size**	**Zones** ^**a**^	**Categorization of the variety**
Guemh	traditional	4	ZN3	Tall DW-type with impurities for stature
Guemh beldi	traditional	8	**ZN1, ZN2,** ZN3	No assignment. Great heterogeneity among populations
Guemh khel	traditional	1	ZN3	Short DW-type with impurities for stature
Guemh lhmer	traditional	9	**ZN1,** ZN2	No assignment. Great heterogeneity among populations
Karim	modern	11	ZN1, **ZN2, ZN3**	Short DW type with impurities for spike morphology and stature. One outlier (mainly tall mixture DW_DC_CA)
Krifla beda	traditional	3	ZN4	Tall DW-type including mixtures with DC.
Krifla kehla	traditional	7	**ZN3, ZN4**	Tall DW-type including impurities for stature
Lehjaoui	traditional	1	ZN1	Tall mixture DW_DC
Le'khel	traditional	4	ZN1, **ZN3**	DW-type, with great heterogeneity among populations for stature
Massa	modern	1	ZN3	Short DW-type with impurities for stature
Mezrouba	traditional	3	**ZN3**	DW-type including mixtures of statures
Pedro	modern	1	ZN3	Short DW type with impurities for stature
Technique	traditional	2	ZN1, ZN2	Short DW type including mixtures of statures
Twinssia	traditional	1	ZN3	DW type, mixture of statures
Twinssia kehla	traditional	2	**ZN3**	No assignment. Tall and heterogeneity among populations for spike morphology
Zeriâa	traditional	4	**ZN3**	DW type, including mixtures with DC, and great heterogeneity among populations for stature
Zeriâa kehla	traditional	1	ZN1	Tall DW-type
Zeriâa twila	traditional	1	ZN2	Mixture of statures and DW_DC

Sample size: number of sampled populations.

^a^ zones where populations of the variety have been sampled. In bold when the sample size was more than one in the zone.

**Table 4:** **Occurrence and morphological characteristics of the varieties cultivated in the Atlas Mountains region**

**Variety**	**Status**	**Sample size**	**Zones** ^**a**^	**Categorization of the variety**
Aberyoun	traditional	15	**ZS2, ZS3**	Tall DC-type, including mixtures with DW.
Chgira lbida	traditional	4	**ZS1**	Tall DW-type, including mixtures with DC and impurities for stature
Cocorit	modern	1	ZS1	Short DW-type
Ifermourgh	traditional	28	**ZS2, ZS3, ZS6**	Tall DC-type, including mixtures with DW. One DW outlier
Ilks	traditional	2	**ZS3**	No assignment. Tall mixture DW-DC
Irden taghezaft	traditional	6	ZS3, **ZS4, ZS5**	No assignment. Tall and great heterogeneity among populations for spike type.
Lbida touila	traditional	6	**ZS1**	Tall DW-type, including mixtures with DC and impurities for stature
Tabakhoucht	traditional	3	**ZS2,** ZS4	No assignment. Tall mixture DW-DC. One short outlier.
Taberyount	traditional	6	ZS2, **ZS3,** ZS5	Tall DC-type, including mixtures with DW. One DW outlier (ZS5)
Tamellalt	traditional	9	**ZS1, ZS2, ZS5**	Tall DW-type, including mixtures with DC.
Toumlilt	traditional	20	**ZS1, ZS2, ZS4, ZS6**	Tall DW-type, including mixtures with DC. One short outlier.
Zerbana	traditional	1	ZS4	No assignment. Tall mixture DW-DC

Sample size: number of sampled populations.

^*a*^ zones where populations of the variety have been sampled. In bold when the sample size was more than one in the zone.

Some varieties couldn’t be categorized, since a wide range of compositions were observed across populations of those varieties (Tables 3 and 4). Namely, most varieties of ZN1 (Guehm beldi and Guemh lhmer) were represented by pure or mixed populations with various spike types, including sometimes bread wheat.

#### Plant stature: the visible impact of modern breeding, even within traditional varieties

**Figure 2:**
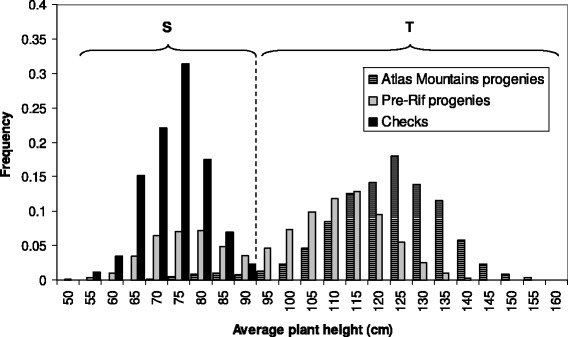
**Frequency distribution of the average height of the checks and of the progenies.** The dashed line delimits the “short” (S) and “tall” (T) statures.

By comparison with the height of the checks, the progenies were classified into one of two statures: tall (T), corresponding to the usual stature of traditional varieties, and short (S), indicative of modern breeding (Figure 2). As for spike type, pure and mixed populations were both observed for stature.

The tall type T was the most frequent within each region and each zone, except in zone ZN2 in the Pre-Rif (Table 2). The Pre-Rif region, where more modern varieties have been recorded [48] and sampled (Table 1), was characterized by a higher proportion of short or mixed populations. In the Atlas Mountains, the vast majority of the sampled populations were made of tall individuals (Figure 1B).

The correspondence between status of the variety (modern *vs* traditional) and plant stature (short *vs* tall) was not strict (Tables 3 and 4). For instance, one population of the modern variety Karim was characterized by tall progenies (Table 3), whereas one population of Tabakhoucht, a traditional variety of the Atlas Mountains mostly contained short individuals (Table 4).

To account for the discrepancy between declared status and stature composition of the population, we will denote as modern-like (resp. traditional-like), a population containing a majority of short individuals (resp. tall individuals).

### Patterns of genetic diversity in *Triticum turgidum*: the INRA collection as a background for the Moroccan sample

One seed per Moroccan population (Moroccan sample, 161 individuals) and Moroccan check (2 varieties) was genotyped with 14 microsatellite loci, as well as 495 samples from a large collection of wild and domesticated subspecies of *Triticum turgidum* (INRA collection)*.*

#### Levels of genetic diversity

In total, 335 alleles were detected for the 14 microsatellite loci over the 658 individuals. The loci showed highly different levels of diversity, from locus Xgwm257 (5 alleles and *H*_*e*_ = 0.327) to Xgwm285 (47 alleles and *H*_*e*_ = 0.870 Table 5)

**Table 5:** **Description and summary statistics of the 14 microsatellite loci used in the present study**

**Locus**	**Chromosome location**	**Allele size range**	**Number of alleles**	***H***_***e***_
Xgpw7577	1B	214-235	9	0.440
Xgwm312	2A	184-291	42	0.785
Xgwm257	2B	180-194	5	0.240
Xgwm374	2B	191-232	19	0.327
Xgwm413	2B	84-123	15	0.850
Xgwm2	3A	211-257	19	0.451
Xgwm285	3B	207-336	47	0.870
Xgwm601	4A	144-176	16	0.763
Xgwm495	4B	161-189	17	0.857
Xgwm234	5B	216-262	26	0.841
Xgwm193	6B	166-206	23	0.859
Xgpw2103	7A	222-300	39	0.862
Xgwm297	7B	146-190	26	0.847
Xgwm537	7B	200-250	32	0.869

*H*_*e*_: expected heterozygosity in the whole sample.

.

Among the subspecies of *Triticum turgidum* from the INRA collection, the wild form ssp. *dicoccoides* was the most diverse, followed by the primitive domesticated form ssp. *dicoccum*. The subspecies ssp. *carthlicum* and ssp. *polonicum* were the less diverse, while ssp. *turgidum* and ssp. *durum* showed intermediate values (Table 6). Within ssp. d*urum*, we further distinguished a set of 161 varieties registered after 1950 (see Methods). When computed only over this set, the diversity of ssp. *durum* decreased to *H*_*e*_ = 0.496 and *R*_*s*_ = 4.53.

**Table 6:** G**enetic variability estimates in the subspecies of** ***Triticum turgidum*** **from the INRA collection and in the Morroccan sample**

**Subspecies of** ***Triticum turgidum*** **and Moroccan sample**	**Sample size**	***N***_***a***_	***H***_***e***_	***R***_***s***_
**ssp.** ***dicoccoides***	53	16.1	0.91^a^	13^a^
**ssp.** ***dicoccum***	94	13.8	0.78^b^	9.78^b^
**ssp.** ***carthlicum***	34	5.1	0.54^ce^	4.69^ce^
**ssp.** ***polonicum***	29	4.8	0.50^de^	4.47^de^
**ssp.** ***turgidum***	33	7.8	0.68^bc^	7.15^bc^
**ssp.** ***durum***	252	8.6	0.56^cd^	5.59^cd^
**Moroccan sample**	161	6.5	0.49^de^	4.57^e^

*N*_*a*_, mean number of alleles per locus; *H*_*e*_, mean expected heterozygosity and *R*_*s*_, mean allelic richness per locus computed for a minimum sample size of 17 diploid individuals per group.

Values with the same letters are not significantly different (P < 0.01).

Only 161 Moroccan individuals have been included in the final data set, due to the identification of 5 *Triticum aestivum* individuals in the original sample.

Overall, the Moroccan sample exhibited the lowest levels of genetic diversity. The level of genetic diversity was not significantly different between the two sampled regions (*H*_*e*_ = 0.48, *R*_*s*_ = 4.69 for the Pre-Rif and *H*_*e*_ = 0.39, *R*_*s*_ = 3.45 for the Atlas Mountains, *P* > 0.01). Most alleles identified in Morocco were included in the allele pool of the INRA collection: only 4 private alleles were detected among the 91 identified. These four alleles were present in the Pre-Rif samples at loci Xgpw7577, Xgwm312, Xgwm495 and Xgwm537, with a frequency ranging from 0.3% to 1.86%.

#### Genetic structure of the INRA collection and its relationship with the Moroccan sample

Our aim was to describe the position of the Moroccan landraces in the background of durum wheat domestication history. For that purpose, we needed first to describe the genetic structure of the INRA collection with respect to its taxonomic and historic subdivisions: The domesticated subspecies of the INRA collection were analyzed using DAPC, a method that identifies clusters of genetically close individuals within a data set [49]. We then projected the Moroccan sample and the checks as supplementary individuals on the obtained clustering.

**Figure 3:**
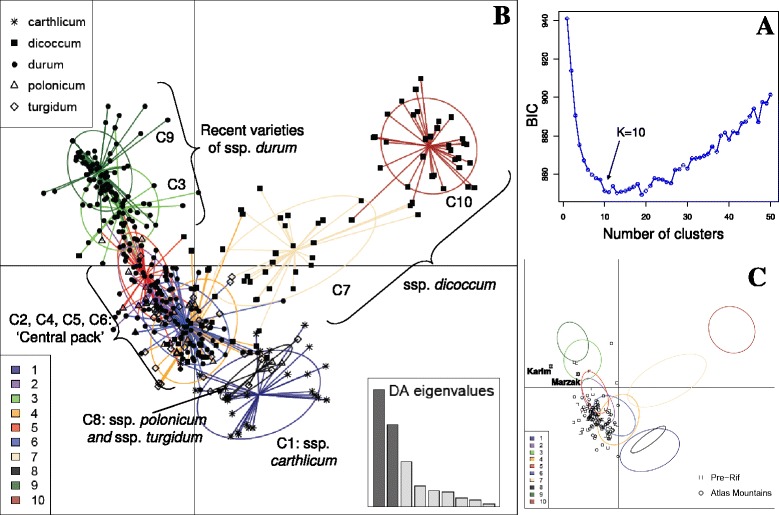
**Results of DAPC applied to the** ***Triticum turgidum*** **collection and to the Moroccan sample. A**. Bayesian Information Criteria (BIC) for increasing values of the number of clusters. The chosen number of clusters was *K* = 10. **B**. Scatterplot of the first two principal components of the DAPC on the collection of domesticated *T. turgidum*. Individuals are represented by symbols according to their subspecies of origin. Numbers, colours and inertia ellipses identify the clusters. The bottom-right inset shows the eigenvalues of the 9 principal discriminant functions. **C**. Scatterplot of the projection of the Moroccan individuals on the genetic clusters identified by DAPC on the collection of domesticated *Triticum turgidum*. The clusters are represented by their inertia ellipses.

The choice of the optimal number of clusters (*K*) was tricky since there was no clear and repeatable value of *K* for which the Bayesian Information Criteria (BIC) value was lowest. Practically, we chose *K* = 10, a value from which the BIC decreased or increased only by negligible amounts in many runs of the analysis (Figure 3A). Note that slightly different values of *K* provided comparable summaries of the data and thus similar qualitative conclusions. Here, we only present the result of one run of DAPC (denoted as the reference run in the following) as well as the repeatability of the obtained clustering based on 15 additional runs with K = 10.

Among the 10 clusters identified in the reference run (Table 7, Figure 3B), three corresponded both to a homogeneous composition regarding the taxonomical position and to a relatively high average membership probability: a cluster with all but one individuals of ssp. *carthlicum* (C1), a cluster only made of ssp. *dicoccum* individuals (C10), and a cluster only made of ssp. *durum* (C9). Three more clusters corresponded to a relative homogeneous composition regarding the taxonomical position and/or to a relatively high average membership probability, although to a lower extent: a cluster almost only made of ssp. *dicoccum* individuals (C7), a cluster almost only made of ssp. *durum* (C3), and a more atypical cluster, including accessions of ssp. *polonicum* and ssp. *turgidum* and well distinguished from the others (C8). Finally, four clusters (C2, C4, C5 and C6) were a mixture of two to four different subspecies, and occupied the central part of the graph. This group of four clusters will be denoted as the “central pack” in the following.

**Table 7:** **Results of DAPC applied to the collection of domesticated** ***Triticum turgidum***

**Subspecies**	**C1**	**C10**	**C7**	**C2**	**C4**	**C5**	**C6**	**C8**	**C3**	**C9**
***carthlicum***	33							1		
***dicoccum***		38	30		18	2	5		1	
***polonicum***				10	10	2	4	3		
***turgidum***			1		15	2	7	8		
***durum***				25	2	46	65		32	82
**Total**	33	38	31	35	45	52	81	12	33	82
**average membership probability**	*0.999*	*1.000*	*0.962*	*0.959*	*0.953*	*0.918*	*0.961*	*0.986*	*0.925*	*0.967*
**metagroups**	CARTH	DIC1	DIC2	CP				PT	DURUM	
**repeatability**	0.984	0.998	0.899	0.974				0.978	0.956	

Repartition of the accessions of the different subspecies among the 10 clusters identified by DAPC, and quality measures of the assignments to clusters and metagroups.

C2, C4, C5, C6 are the 4 genetic clusters belonging to the ‘central pack’.

*Metagroups* have been defined based on the repeatability of the groupings in 15 additional runs (see text). The repeatability corresponds to the average proportion of the individuals from the original metagroup that were assigned together to a similar metagroup in the repetitions.

Interestingly, the two homogeneous clusters of ssp. *durum* C3 and C9 included almost exclusively varieties registered from the 50ies, whereas all landraces were included in the central pack (data not shown).

Overall, the 15 additional runs gave similar qualitative results. The 6 clusters not belonging to the central pack were easily recognizable in the new clusterings, even if some were subdivided or merged in a few runs. A central pack was always present but with different subdivisions and a different number of clusters among runs. We thus decided to group some clusters into “metagroups” and evaluated the repeatability of these metagroups as the average proportion of individuals of the metagroup of the reference run, that was assigned to a similar metagroup in the new runs, or that clustered altogether to another metagroup in case of merging. We obtained a high repeatability score (>0.899, Table 7).

The Moroccan genotypes projected as supplementary individuals were mainly assigned to the four clusters of the central pack, with a few (11) assigned to the C9 cluster containing the most recent varieties (Figure 3C). The Moroccan plants assigned to the C9 cluster were mainly from modern varieties or modern-like populations. These assignments contrasted with the high frequency of spike-types indicative of ssp. *dicoccum* (Table 2). The unstability of the strucure of the central pack prevented us to be more precise on the historical position of the Moroccan landraces.

### Geographical patterns of genetic diversity of durum wheat in Morocco

#### Levels of genetic diversity

Within regions, the zones differed only slightly for the amount of genetic diversity measured as *H*_*e*_ and *R*_*s*_: we detected 5 significant differences out of 42 tests. In the Pre-Rif Region, only ZN4 had a lower allelic richness compared to the three other zones. In the Atlas Mountains, ZS3 (at the highest elevation) and ZS5 exhibited the lowest level of genetic diversity, whereas ZS2 was the most genetically diverse zone. As expected for a selfing species, genotypes were highly homozygous. A few accessions were heterozygote at one locus at least: they represented 13% of our sample in both regions (Table 8).

**Table 8:** **Genetic diversity estimates for the Moroccan sample of durum wheat**

**Regions**	**Zones**	**Sample size**	***N***_***a***_	***H***_***e***_	***R***_***s***_	**Nb Het**
**Pre-Rif**	**ZN1**	13	3.5	0.412^a^	2.75^ab^	3
	**ZN2**	10	3.1	0.418^a^	2.67^a^	1
	**ZN3**	32	4.2	0.449^a^	2.89^ab^	3
	**ZN4**	5	2.1	0.411^a^	2.07^b^	1
**Atlas Mountains**	**ZS1**	15	2.6	0.367^ab^	2.23^a^	0
	**ZS2**	22	3.3	0.446^a^	2.53^a^	6
	**ZS3**	27	3	0.334^b^	2.19^ab^	3
	**ZS4**	8	2.5	0.426^ab^	2.40^a^	0
	**ZS5**	6	1.8	0.314^ab^	1.77^b^	1
	**ZS6**	23	3	0.356^ab^	2.25^ab^	3

*N*_*a*_, mean number of alleles per locus; *H*_*e*_, mean expected heterozygosity and *R*_*s*_, mean allelic richness per locus computed for a minimum sample size of 5 diploid individuals per group. Nb Het, number of heterozygote genotypes.

Values with the same letters are not significantly different (*P* < 0.01). Pairwise tests were performed within regions.

#### Genetic structure among geographic groups and among varieties

A hierarchical analysis of molecular variance (AMOVA) was performed with zones nested within regions. It detected significant differentiation both between regions (F_CT_ = 0.146, *p* < 0.001) and between zones within regions (F_SC_ = 0.171, *p* < 0.001). Most of the genetic variance was observed within zones (70.7%) whereas the remaining variance was almost equally partitioned among zones within regions (14.7%) and among regions (14.6%). One hundred and twelve different genotypes were identified in the Moroccan data set. Only three were shared between the two regions: two were identified in modern-like populations and encompassed respectively 2 and 4 different varietal names (different between regions); the third was present in ten Atlas traditional populations (belonging to 4 different varieties) and in one population described as Krifla beda, a traditional variety of the Pre-Rif. We applied DAPC to the Moroccan sample: individuals from different regions were overall assigned to different clusters. Only 9 individuals were assigned to clusters made of individuals from another region (Additional file 1: Figure S1 and Additional file 2: Table S2). Six individuals from the Pre-Rif were assigned to the 4 Atlas-Mountains clusters (5.7% of the individuals of these clusters). Three individuals from the Atlas Mountains were assigned to the 5 Pre-Rif clusters (5.3% of the individuals of these clusters). These exceptions were in accordance with the pattern of shared genotypes between regions described above. We can thus confidently go on by analysing separately the two regions.

We computed two kinds of *F*_*ST*_ values between zones: one used the microsatellite genotypes of the samples; the other used as a genotype the name of the variety of origin of the sample. In the Pre-Rif, microsatellite-*F*_*ST*_ values were roughly higher between more distant zones. All zones were significantly differentiated from each other, except ZN2 and ZN3 which, relative to the others, were also similar for the frequency of both short – modern-like – statures and spike types (Table 2, Table 9). The pattern was similar for *F*_*ST*_ values based on variety names, even if the relationships was not stringent.

**Table 9:** **Pairwise differentiation indices between zones of the Pre-Rif**

**Studied areas**	**ZN1**	**ZN2**	**ZN3**	**ZN4**
ZN1		**0.165**	**0.185**	**0.256**
ZN2	**0.136**		NS	**0.170**
ZN3	**0.149**	**0.059**		**0.160**
ZN4	**0.318**	**0.281**	**0.150**	

Above diagonal: pairwise genetic differentiation index (*F*_*ST*_). Below diagonal: *F*_*ST*_ based on variety names (see Methods). Only values significant at the 0.05 levels are reported. NS: non significant.

In the Atlas Mountains, two genetically distinct groups emerged from pairwise microsatellite-*F*_*ST*_ comparisons between zones (Table 10). One group included ZS1, ZS2, ZS4 and ZS5 and the other ZS3 and ZS6. Indeed, zones of the first group were all significantly differentiated from zones of the other group. Moreover, no significant differentiation between zones was found within each group. This pattern mirrored the different spike-type frequencies measured in these two groups of zones (Table 2). By contrast, it was not coherent with the *F*_*ST*_ values based on variety names. For instance, a relatively high differentiation based on variety names was detected between ZS3 and ZS6. This inconsistency suggests that the varietal taxonomy is a poor indicator of genetic differentiation.

**Table 10:** **Pairwise differentiation indices between zones of the Atlas Mountains**

**Studied areas**	**ZS1**	**ZS2**	**ZS3**	**ZS4**	**ZS5**	**ZS6**
ZS1		NS	**0.243**	NS	NS	**0.150**
ZS2	**0.160**		**0.204**	NS	NS	**0.172**
ZS3	**0.265**	**0.205**		**0.215**	**0.306**	0.014
ZS4	**0.176**	NS	**0.257**		NS	**0.136**
ZS5	**0.184**	**0.170**	**0.262**	NS		**0.196**
ZS6	**0.439**	**0.301**	**0.332**	**0.443**	**0.530**	

Above diagonal: pairwise genetic differentiation index (*F*_*ST*_). Below diagonal: *F*_*ST*_ based on variety names (see Methods). Only values significant at the 0.05 levels are reported. NS: non significant

**Figure 4:**
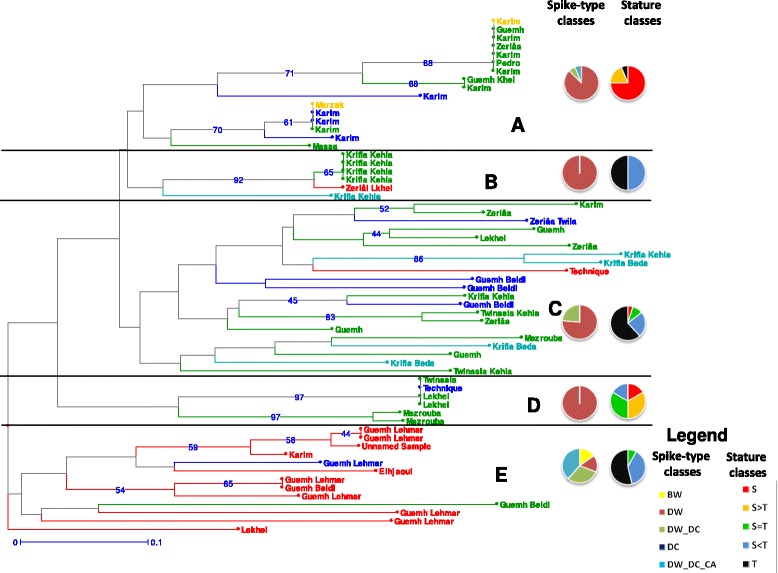
**Neighbour joining tree among Pre-Rif populations based on genotypic data.** Populations are labelled by the name of the variety they belong to, and coloured according to their zone of origin: red for ZN1, blue for ZN2, green for ZN3 and light blue for ZN4. The Moroccan checks are coloured in yellow. Horizontal lines separate the five clusters discussed in the text. Each cluster is identified by a letter. The pie charts depict the composition of each cluster in term of spike-type and stature classes of populations. Bootstrap values are reported when higher than 40%.

**Figure 5:**
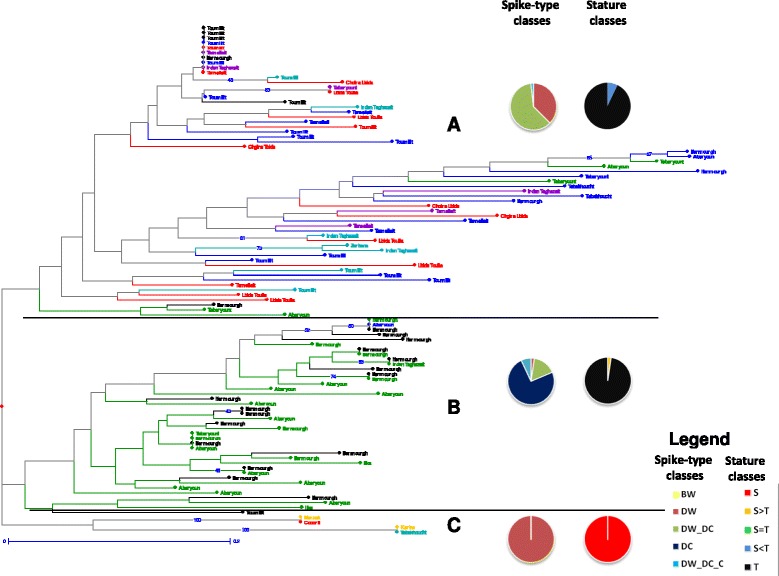
**Neighbour joining tree among Atlas populations based on genotypic data.** Populations are labelled by the name of the variety they belong to, and coloured according to their zone of origin: red for ZS1, blue for ZS2, green for ZS3, light blue for ZS4, violet for ZS5 and black for ZS6. The Moroccan checks are coloured in yellow. Horizontal lines separate the three clusters discussed in the text. Each cluster is identified by a letter. The pie charts depict the composition of each cluster in term of spike-type and stature classes of populations. Bootstrap values are reported when higher than 40%.

Within each region, we built a neighbor joining tree based on the genetic dissimilarity between multilocus genotypes. In both regions, there was no strict equivalence between variety name and multilocus genotype (Figures 4 and 5). Although some samples belonging to the same variety could be genetically identical or very close (e.g. Krifla kehla in the Pre-Rif Region, cluster B, Figure 4), some were very distant from each other (e.g. Guemh that appeared in cluster A and C, Figure 4). Reciprocally, some varieties appeared undistinguishable from each other: for instance, individuals belonging to the traditional varieties Ifermourgh and Aberyoun grown in the Atlas Mountains were intermixed (Figure 5, cluster B).

High bootstrap values were only found for some terminal clusters, and the deep branching patterns were not well supported. However, some tendencies deserved to be highlighted, as they were partly related either to the status of the varieties or to some of their morphological categorization (Tables 3 and 4). In the Pre-Rif, five clusters could be distinguished (Figure 5). One cluster (E) grouped populations from ZN1, which often included different spike-types or statures. The four other clusters distinguished modern-like populations (namely, made of a majority of short progenies, clusters A and D), from traditional-like populations (namely, made of a majority of tall progenies, clusters B and C). The frequency of short or tall statures in the population was thus more discriminating than the declared status for interpreting the topology of the tree (Tables 3 and 4); for instance, one traditional-like population of the modern variety Karim appeared in cluster C.

In the Atlas Mountains, three clusters could be identified. One cluster included modern varieties or modern-like populations (C). The two others reflected a combination of the zone of origin (ZS3 and ZS6 for cluster B *vs*. ZS1, ZS2, ZS4 and ZS5 for cluster A) and of the spike-type class of the population (DC-type for cluster B *vs* DW-type for cluster A) (Figure 5). The correspondence between clusters and zone or spike-type was not strict, but this partitioning remarkably mirrored the one inferred from spike-type frequencies and from pairwise *F*_*ST*_ values between zones (Table 2, Table 10).

## Discussion

### Morocco in the light of wheat history: a diversified morphological basis contrasting with a relatively reduced and unspecific neutral diversity

We identified in the Moroccan sample individuals and whole populations as bread wheat, even if these populations had been explicitly designated by farmers as durum wheat. In some countries, farmers consciously grow mixtures of durum and bread wheat [36,45] but such practices have not been mentioned here. We lack information to understand our observation. It could be partly explained by the fact that bread wheat was introduced only a century ago in the area and is not yet well known by the farmers. One of the consequence of these mixtures it that they could allow interspecific hybridization, which has already occurred in wheat history [36], and may lead to original phenotypes and/or genotypes. The contribution of interspecific hybridization to traditional varieties diversity has for instance been evidenced between Asian (*Oryza sativa*) and African rice (*Oryza glaberrima*) in different African countries i.e. occurrence of varieties described as ‘hybrid’, [50]. Interestingly, the ZN1 zone where bread wheat was identified appeared in majority in a distinct cluster in the NJ tree (even if no bread wheat individual was included in the genetic dataset, cluster E, Figure 4). But this is an insufficient indication to conclude on interspecific hybridization.

Plants with ancestral spike shape of the dicoccum type were observed within Moroccan landrace populations but they were free-threshing at maturity. Moreover, they did not cluster with the specific ssp. *dicoccum* clusters of the collection (C7, C10, Figure 3C). In other countries, free-threshing dicoccum landraces have been described, as well as durum landraces partially introgressed with dicoccum genes resp. in Yemen and Ethiopia, [45]. We have no information on present-day emmer wheat (ssp. *dicoccum*) cultivation in Morocco, but it was documented during the first part of the twentieth century [45]. Here, the dicoccum-like landraces could result from recombination between different ancestral gene pools. Whatever their origins, the reason why such forms are preferentially cultivated in some zones (for instance for their local adaptation or for some particular uses) and whether they relate to more difficult post-harvest processing deserve further investigation. According to the interview data [48], even though farmers identified some varieties as easier to thresh, these varieties were most often the modern ones. Using direct measurements, variability has already been evidenced for threshing efficiency within a diversified sample of free-threshing durum wheat landraces [35]. Such measurements could be applied to our sample.

As a whole, the high morphological diversity in the Moroccan tetraploid wheat sample did not correspond to a high and/or original genetic diversity relative to the *Triticum turgidum* INRA collection. Moroccan genetic diversity was relatively reduced and similar to that of the group of modern varieties from the INRA collection registered since the 1950ies. Such result is however not surprising considering the restricted geographical range of our sampling. Oliveira et al. [41] also identified North-West Africa as the Mediterranean region with the least genetic diversity for durum wheat. The Moroccan genetic diversity has also been shown to be lower than for Syrian landraces [46]. Moreover, except for ssp. *carthlicum* which is supposed to result from hybridization with bread wheat, [36], the free-threshing subspecies did not correspond to genetically distinguishable entities also as previously shown, [41] and were combined in a metagroup with no clear or consistent composition (metagroup CP, Table 7, Figure 3B): almost all Moroccan individuals were assigned to that metagroup. More information on the historical origin of Moroccan durum wheat diversity appears then difficult to gather with our sample, considering the complex and still unresolved history of durum wheat across the Mediterranean basin long period of cultivation, repeated introductions, and gene flow between the different origins, [41].

### Geographical and varietal differentiation in Morocco: large-scale patterns but loose local barriers to gene flow and mixing

Chentoufi et *al.* [48] inventoried the varieties grown by farmers and showed that except for modern varieties, no names were shared between the two study regions. Such differentiation was supported by our genetic results. Indeed, the Pre-Rif and the Atlas Mountains samples were strongly differentiated from each other. We thus have two regions that kept their originality, which confirmed the large-scale geographic structure detected by Kehel et al. [46]. Such high differentiation is not surprising given that these two distant regions display very different environmental, cultural and agronomic conditions. This is also a common result in the study of diversity of traditional varieties, as exemplified by sorghum [18]. However, in some countries, studies have shown a lack of geographical structure despite a strong environmental heterogeneity, a pattern attributed to large-scale seed exchange and continuous seed introduction e.g. barley in Ethiopia, [51]. This is apparently not (yet) the case in Morocco.

Within regions, although variety names were rather localized [48], only a few patterns emerged from the genetic and morphologic characterization, as if neither distance nor different variety names constituted consistent barriers to genetic exchange. Moreover, 48% of populations combined different statures and spike types (40% when considering only spike type); some populations even had a radically different composition than the other populations within their varieties. Such inconsistencies have been evidenced recurrently, at various levels of investigation e.g. [52]. They illustrate the limits of the use of variety names to assess genetic and morphological diversity [53] and to elaborate sampling strategies for the conservation of genetic resources [28].

Besides this, we can point out some mechanisms potentially involved in these patterns (and lacks of pattern) and open tracks of future research to decipher them.

First, the large proportion of mixed populations (on the base of spike type and stature) suggests the occurrence of intermixing and genetic exchanges between varieties with different morphological composition (DC or DW spike-types, and short or tall statures). These exchanges might have different sources: unconscious mixing in threshing areas used by different farmers and for different varieties, unreliability of seed exchange networks (e.g. markets providing seed lots that do not correspond to the declared names) [54], limited farmers’ interest for durum wheat cultivation and seed production. These mixtures create strong opportunities to generate diversity through cross-fertilization (heterozygote genotypes were observed) and recombination. One question of interest is whether these mixtures will homogenize the pool of traditional varieties, or whether mechanisms such as human practices for instance choice of less admixed plant for seed production [55] or divergent environmental pressures for instance, when populations of a given landrace are grown in different environments, [21,56] will maintain some differentiation between them.

Second, the taxonomy of varieties and how farmers experience this taxonomy is questioned by our results. Indeed, our morphological and genetic data suggest that names of some varieties are interchangeable (Table 4 and Figure 5), for instance Ifermourgh and Aberyoun in the Atlas Mountains. By contrast, some names or groups of names carry information on genetic distinctiveness: for instance Krifla Kehla in the Pre-Rif (cluster B, Figure 4) and the two differentiated groups of traditional varieties in the Atlas Mountains (i.e. landraces corresponding to DC and DW spike types). (i) First, if these names exist, there is probably a reason to which we do not have any access yet. We need to understand which criteria farmers use to distinguish and name their traditional varieties. The interviews described in Chentoufi et al. [48] only identified criteria distinguishing modern and traditional varieties. The use of less oriented protocols should allow the expression of the plants’ and populations’ characteristics actually recognized and used by the farmers themselves [57]. (ii) Second, if there are criteria, the question is then whether all the farmers still know and use them. Interestingly, the interviews have shown a rapid recent renewal of varieties at the farm level in the Pre-Rif region, possibly for climatic reasons (crop failures due to limited rainfall) [48]. Such phenomenon has also been observed for barley in this region [26]. Farmers from the Pre-Rif region regularly have to outsource their seeds and often grow different varieties over years: this may reduce the knowledge associated with each variety and allow mixing and errors, depending on the reliability of the seed exchange networks. In this region, except for a few groupings, we could not discriminate traditional varieties based on their genotypes. In contrast, the two genetically and morphologically well-differentiated groups of varieties in the Atlas Mountains coexist even when cultivated in the same area (ZS6). This suggests that here, farmers’ practices maintain these two groups well-differentiated. Such variable importance given to a crop in different areas, associated with more or less frequent crop failure and seed renewal has been evidenced by Samberg et al. [24] for barley in Ethiopia. Investigations on the practices of selection and choices, in the field or during seed exchange, are required to understand these contrasting patterns.

Finally, we have shown that the two well-differentiated groups of varieties of the Atlas Mountains partially overlapped with the two geographic groups of zones (ZS3-ZS6 *vs* the four other zones). Assessing the relative effect of local adaptation and gene flow would help understanding this pattern. For instance, local adaptation may arise from different selective pressures along the altitudinal gradient in the Ziz valley [20]. Reciprocally, informal discussions suggest that farmers from ZS3 regularly renew their seeds from the much lower altitude zone ZS6 following crop failures in cold years: such preferential exchanges might explain the differentiation of these two zones relative to the others.

### The signature of the incorporation of modern varieties into the traditional network

Modern varieties have been introduced in Moroccan agrosystems in the 1970ies, during the Green revolution. Chentoufi et al. [48] showed that they were particularly present in the Pre-Rif region and that they were submitted to the same agricultural practices than the traditional varieties [48]. The present study showed that the cultivation of modern varieties resulted in gene flow between modern and traditional varieties. Indeed, some populations of traditional and modern varieties combined short (indicative of modern breeding) and tall plants (illustrating the double use of traditional varieties, for straw and grain). Moreover, identical, or similar genotypes were shared between varieties of different status.

Such exchanges have occurred repeatedly in cultivated plants whatever their mating system. For instance, introgression from modern into traditional varieties has been evidenced in maize (outcrossing) and barley (selfing) [58,59]. In Mexico, the introduction of modern varieties of maize into traditional agrosystems gave rise to what is called “creolized” varieties [23]. In these cases, landraces often maintained their genetic identity, and an increase in genetic diversity could even be observed at the agrosystem level due to the introduction of new alleles. However, whenever the pressure of modern varieties becomes too strong, i.e. when they become more prevalent than the traditional ones, an overall loss of diversity is expected [60]. In the Pre-Rif region, the surveys performed by Chentoufi et al. [48] revealed that the cultivation of modern varieties increased in the previous years, and that traditional varieties even disappeared from some villages. Together with our results on introgression, this claims for measures dedicated to the conservation of the traditional varieties.

## Conclusions

Our study, together with the interviews reported in Chentoufi *et al.* [48], provides an essential first assessment of the varietal, phenotypic and genetic diversity present in Moroccan agroecosystems. Despite our limited level of investigation (namely large-scale geographical sampling, with only a single genotyped individual per population), we interpreted our results in the light of data collected from farmers’ interview, and identified a number of investigation tracks to arrive at a good comprehension of the processes generating diversity in these traditional agrosystems, and of the factors that might affect their future evolution.

Following these different tracks requires to concentrate investigations at a small scale, potentially different for each question and to integrate contributions from different disciplines [24]: consideration of the socio-cultural context [16], investigation on the seed exchange practices [27,54], sampling simultaneously intrapopulation and interpopulation diversity for some landraces [19], considering the genetic diversity present on local markets [24,26], investigating more detailed selection, threshing or other practices [55,61], and collecting precise environmental variables on the study sites [21].

Climatic, economic and political contexts have been changing rapidly in traditional agrosystems during the last few decades following for instance the development of communication routes and agricultural policies, as described for Ethiopia in Samberg et al. [27]. Understanding the current dynamic of traditional agrosystems, by reciprocal learning between farmers and scientists [4], is the necessary step for conservation and use of local diversity in the face of these changes.

## Methods

### Study areas and sample collection

Chentoufi et al. [48] surveyed the durum varieties cultivated in two contrasted agro-ecological regions: the Pre-Rif region in the North of Morocco (medium mountains, rain-fed agriculture) and the oases of the Atlas Mountains (marked altitudinal gradient, irrigated agriculture) in the South-East. The Pre-Rif area is located at the interface between plains were agriculture has been strongly modernized and mountains were traditional agriculture still prevails. It was subdivided into four zones on the basis of geography. The oases of the Atlas Mountains remained more isolated from the other parts of the countries due to transport difficulties in mountainous areas; this region subdivided into six zones on the basis of altitudinal range and geography (Figure 1). Each cultivated variety was identified by the name provided by the farmer: 19 different names were recorded in the Pre-Rif from 163 farmers’ interviews, and 14 in the Atlas Mountains from 101 farmers’ interviews. More details on the study areas and on the varietal survey can be found in Chentoufi et al. [48]. Varieties were classified as traditional or modern by the farmers. Because a given variety can be grown in different farms, we denote by “population” the particular seed lot of a variety actually sown by a given farmer. Farmers don’t grow multiple varieties in the same field: a field then corresponds to a population.

Based on these results, we followed two different sampling strategies in the two regions. In the Pre-Rif region, we adopted a sampling strategy allowing the collection of as many as possible of the wheat varieties. In total, 65 durum wheat populations were sampled. These populations corresponded to 18 different varieties: 17 identified during the initial survey of Chentoufi et al. [48] and one additional variety identified during our collecting trip. In the oases of the Atlas Mountains, we sampled one population from each of the surveyed farmers. In total, we sampled 101 wheat populations in this region, corresponding to 12 different varieties (Table 1, Figure 1). No names were shared between the two regions.

For each of the 166 wheat populations, 30 spikes from 30 randomly chosen plants were harvested in the farmer’s field.

### Morphological characterization

During the 2011/2012 growing season, 20 seeds from each individual spike were sown in an ear-row design (1 m length and 0.3 m between rows) at the experimental station of the IAV Hassan 2 (Rabat, N 33°58’35”, W 6°51’59”, 50 m above sea level). Two Moroccan durum wheat modern varieties (Karim and Marzak, seeds provided by Moroccan National Institute for Agricultural Research) were used as checks to account for potential environmental variation at the field station: 74 rows of Karim and 39 of Marzak were sown across the experiment (20 seeds per row).

In the following, we will denote as a progeny the maternal family corresponding to the seeds from a given spike.

We recorded two key morphological traits that are informative on durum wheat history, namely spike shape and plant height. We classified progenies into four categories of spike: (i) dicoccum-like spikes, *i.e.* flat ear with only three flowers per spikelet (denoted as DC); this corresponds to the shape expected for plants carrying the *q* allele of the *Q* gene [34] but note that these spikes have not systematically a hulled phenotype at maturity; (ii) lax and flat spikes with long rachis and elongated rachilla internodes. Because this morphology can be indicative of *T. turgidum* ssp. *carthlicum* or of bread wheat *T. aestivum*, species status was checked by flow cytometric analysis for all progenies of the populations where it was observed protocol adapted from [62]. Hexaploid progenies were classified as bread wheat (denoted as BW) and tetraploid ones as carthlicum type (denoted as CA); and (iii) classical square-shaped durum wheat spike type (compact spike with 5 flowers per spikelet, denoted as DW, corresponding to the shape of *Q* allele-carrying plants [34]). These four categories (DC, BW, CA and DW) will be denoted as spike type in the following.

Average plant height was recorded for each progeny by averaging 3 measures taken across the row (Figure 2). For the checks, it ranged from 55 to 90 cm; this range of values was considered as representative of modern varieties where dwarfing genes have been introduced [42]. In the Moroccan sample, the distribution was clearly bimodal, the first mode matching the checks’ distribution. The upper limit of the checks’ distribution was then used to assign each progeny to one of two plant statures: short (S, average height less or equal to 90 cm, possibly carrying dwarfing genes), or tall (T, average height larger than 90 cm).

### DNA extraction and genotyping procedure

For each population composed of thirty collected spikes, we randomly picked one spike. One seed per spike and one for each Moroccan check were sown for DNA extraction.

Genomic DNA was extracted from 100 mg of fresh leaf tissue of three weeks old seedlings. The extraction was performed following a protocol adapted from Tai and Tanksley (1991). DNA quality was checked on agarose gel and the DNA concentration was estimated using spectro-fluorometry. Five samples of the Pre-Rif belonged to progenies identified as hexaploid wheat (BW type, see above) and were excluded from the genetic analysis. Thus, 163 Moroccan individuals were included in the final data set: 161 populations (Moroccan sample) and two checks.

In addition, we analyzed a set of 495 DNA samples of genotypes from a large collection representing different subspecies of *Triticum turgidum* conserved at INRA (INRA collection). It included 53 accessions of the wild ancestor *T. turgidum* ssp. *dicoccoides*, 94 accessions of ssp. *dicoccum*, 34 of ssp. *carthlicum*, 29 of ssp. *polonicum*, 33 of ssp. *turgidum* and 252 of ssp. *durum* subsets of this sample have been analyzed in [39,63]. Available information on the ssp. *durum* accessions allowed distinguishing among them 69 landraces from Lebanon, Jordan, Syria and Turkey and 161 varieties of durum wheat registered after 1950 (122 from France and 39 from other countries).

Fourteen nuclear SSR markers used in previous assessments of durum wheat diversity and mapping to a single, genome specific, locus sequences available at http://wheat.pw.usda.gov/GG2/index.shtml, [38,61] were selected. Polymerase chain reaction (PCR) amplification was performed in a total volume of 20 μl containing 50 ng of genomic DNA, 2 nM MgCl_2_, 0.2 mM of each dNTP, 0.5 units of Taq DNA polymerase and 1-4 pM of each primer. The forward primer was labeled with one of the three fluorophores (6FAM, NED or HEX). PCR was carried out as follows: after 5 min at 94°, 35 cycles were performed with 30 s at 94°C, 30 s at either 55 or 60°C (depending on the locus), and 1 min at 72°C, followed by final extension step of 30 min at 72°C. Amplified products were detected on an ABI 3130xl Genetic Analyser (Applied Biosystems, Foster City, CA, USA) and analyzed using the GENEMAPPER V3.7 software (Applied Biosystems).

### Morphological data analysis

Frequencies of spike types and of plant statures among progenies were computed for each population and each zone.

Then, frequencies within populations were used to assign each population to a class. First, for each of the two morphological traits, we denoted as pure populations the populations where all sampled progenies were of the same type (i.e. spike type or stature) with the allowance of one outlier, and as mixed populations the ones combined different types, with at least two progenies of each. For spike types, we defined five classes: (i) BW, corresponding to populations containing bread wheat (*T. aestivum*) individuals. This class included pure populations of bread wheat and mixed populations with a majority of bread wheat progenies associated with *T. turgidum*, (ii) DW and (iii) DC, corresponding to pure populations of respectively DW and DC spike type, (iv) DW_DC, corresponding to mixed populations combining DC and DW spike types, and (v) DC-DW-CA, corresponding to mixed populations including these three spike types. Note that the CA type was always rare within populations.

For plant stature, we defined five classes of populations: S and T, corresponding respectively to pure populations of short or tall progenies, and three kinds of mixed populations (S < T, T = S, and S > T, when respectively the frequency of short progenies was lower than 33%, between 33 and 66%, and higher than 66%).

### Genetic diversity and population structure

The mean number of alleles per locus (N_a_), mean allelic richness Rs, [64], and expected heterozygosity He, [65] were computed using FSTAT 2.9.3.2 software [66] in different groups of the dataset, namely subspecies of *T. turgidum*, whole Moroccan sample, regions within Morocco, and zones within regions. Differences in the amount of genetic diversity between groups were tested by Wilcoxon signed-rank tests, comparing values for the same loci in different groups, using R (R Development Core Team 2008). For these tests, we used the significance threshold *P* = 0.01.

Population structure within the INRA collection of *T. turgidum* was examined by applying the discriminant analysis of principal components DAPC, [49], a multivariate method designed to identify and describe clusters of genetically related individuals. The method relies on allele data transformation using principal component analysis as a prior step to discriminant analysis. DAPC was performed using the adegenet package [67] in R (R development Core Team). DAPC is particularly useful for identifying population structure without assuming a population model that would not be supported in our data set.

We first ran DAPC on the INRA collection, including only the domesticated subspecies of *T. turgidum*. The optimal number of clusters was determined using the *find.clusters* function which implements successive K-means clustering. The rate of decrease of the Bayesian information criterion (BIC) was visually examined, and the number of clusters was determined as the value of *K* above which BIC values decreased only subtly. In order to obtain reliable group membership probabilities, the number of Principal Components (PC) retained to compute the discriminant functions was determined using the function *optim.a.score*, which examines the trade-off between power of discrimination and over-fitting. Here we retained 11 to 13 PC depending on the runs. We then projected the Moroccan genotypes as supplementary individuals onto the discriminant functions and examined their assignment to the different clusters. A DAPC analysis was run on the Moroccan sample alone, using the same process as described for the INRA collection.

Within Morocco, a hierarchical analysis of molecular variance was performed with zones nested within regions, using ARLEQUIN v 3.5 [68]. The significance of the fixation indices and of the variance components was assessed using 10 000 permutations.

Genetic differentiation between zones within region (*F*_*ST*_) was estimated using the method of Weir and Cockerham [69], with the software Genetix V4.05 [70]. To quantify how much zones differed for the varieties they host, we also computed *F*_*ST*_ estimates based on variety names: Namely, each individual was described by its variety name, which was considered as equivalent to a homozygous genotype at a single locus. These *F*_*ST*_ values were compared with the classical *F*_*ST*_ values based on microsatellite genotypes. It is a way to assess the discrepancy between the information provided by the genotype and by the name of the variety of origin of a sample.

In order to depict the genetic relationships between populations, DARwin version 5.0 was used to compute a genetic dissimilarity matrix using the simple matching index [71]. The dissimilarity matrix was then subjected to cluster analysis using the weighted neighbour-joining method to obtain a dendrogram for the Pre-Rif accessions and the Atlas Mountains populations. The uncertainty of the dendrogram structure was assessed with 1000 bootstraps.

## Additional files

Additional file 1: Figure S1. Results of DAPC applied to the Moroccan sample.A. Bayesian Information Criteria (BIC) for increasing values of the number of clusters. The chosen number of clusters was *K* = 9. B. Scatterplot of the first two principal components of the DAPC on the sample of Moroccan populations. Individuals are represented by symbols according to their region of origin. Numbers, colours and inertia ellipses identify the clusters. The bottom-right inset shows the eigenvalues of the 6 first principal discriminant functions. Additional file 2: Table S1. Results of DAPC applied to the Moroccan Sample Repartition of the Moroccan populations from the different regions among the 9 clusters identified by DAPC, and quality measures of the assignments to clusters.
