# Supplementary figures and images for: Towards a comprehensive characterization of durum wheat landraces in Moroccan traditional agrosystems: analysing genetic diversity in the light of geography, farmers’ taxonomy and tetraploid wheat domestication history
Source: BMC Evol Biol. 2014 Dec 21;14:264. doi: 10.1186/s12862-014-0264-2 (PMC4300848; doi:10.1186/s12862-014-0264-2)

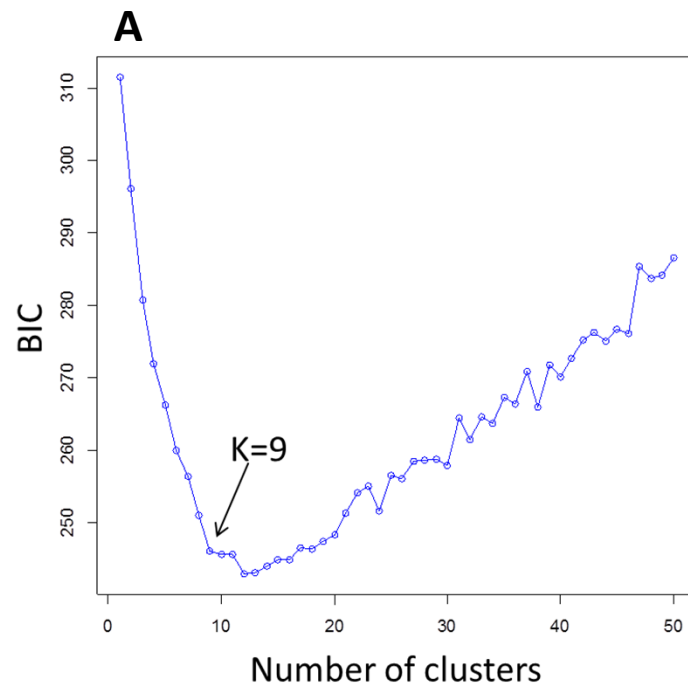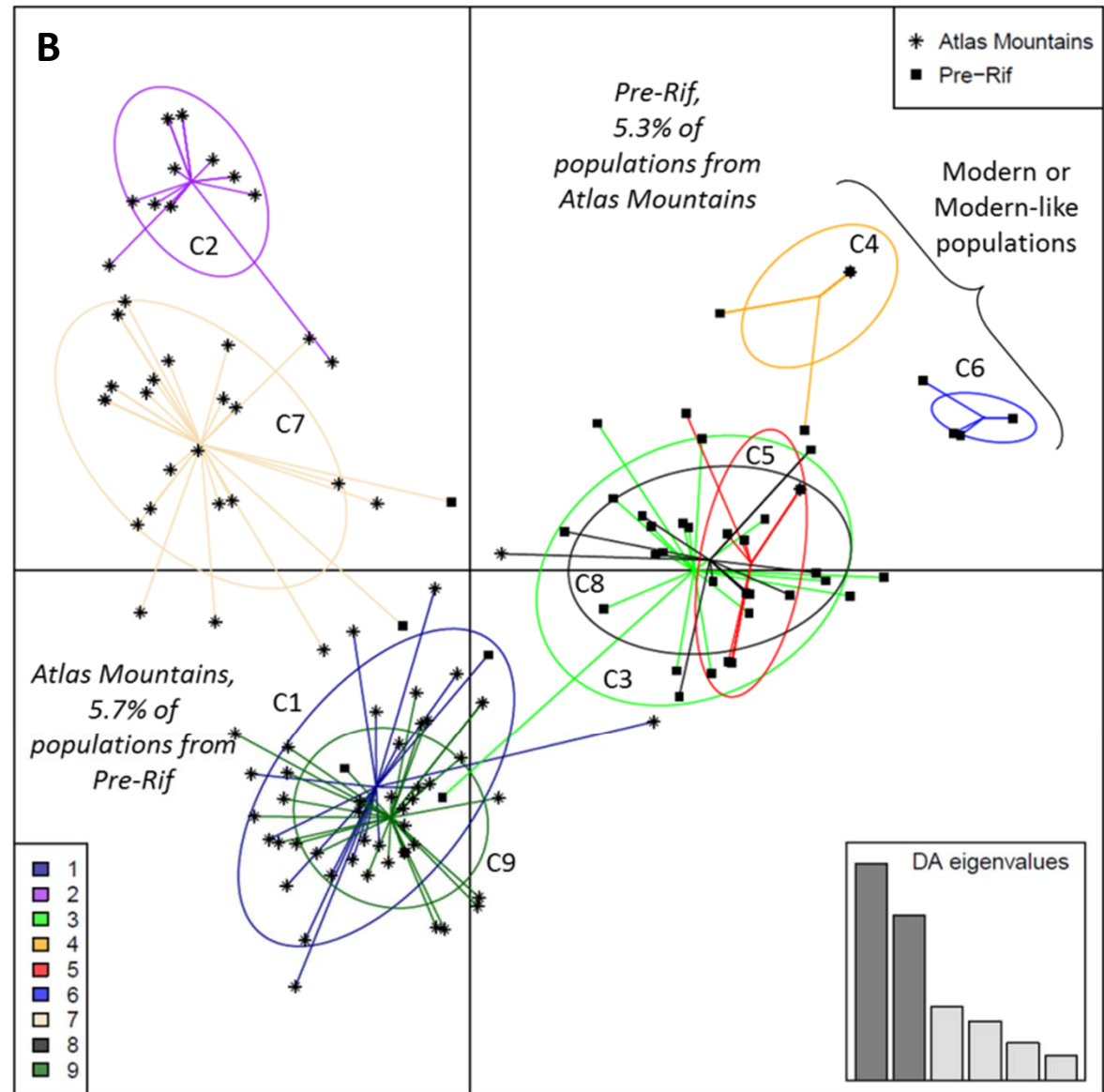

Supplement: Additional file 1: Figure S1. — Results of DAPC applied to the Moroccan sample.A. Bayesian Information Criteria (BIC) for increasing values of the number of clusters. The chosen number of clusters was K = 9. B. Scatterplot of the first two principal components of the DAPC on the sample of Moroccan populations. Individuals are represented by symbols according to their region of origin. Numbers, colours and inertia ellipses identify the clusters. The bottom-right inset shows the eigenvalues of the 6 first principal discriminant functions. [file 12862_2014_264_MOESM1_ESM.pdf]
