# Supplementary material for: Towards a comprehensive characterization of durum wheat landraces in Moroccan traditional agrosystems: analysing genetic diversity in the light of geography, farmers’ taxonomy and tetraploid wheat domestication history
Source: BMC Evol Biol. 2014 Dec 21;14:264. doi: 10.1186/s12862-014-0264-2 (PMC4300848; doi:10.1186/s12862-014-0264-2)
Supplement: Additional file 2: Table S1. — Results of DAPC applied to the Moroccan Sample Repartition of the Moroccan populations from the different regions among the 9 clusters identified by DAPC, and quality measures of the assignments to clusters. [file 12862_2014_264_MOESM2_ESM.docx]

**Supplementary Table: Results of DAPC applied to the Moroccan Sample**

| **Region** | **C1** | **C2** | **C7** | **C9** | **C3** | **C5** | **C8** | **C4** | **C6** |
| --- | --- | --- | --- | --- | --- | --- | --- | --- | --- |
| **Atlas Mountains** | 16 | 16 | 27 | 39 |  | 2 |  | 1 |  |
| **Pre-Rif** | 1 |  | 2 | 3 | 15 | 11 | 12 | 9 | 7 |
| **Total** | 17 | 16 | 29 | 42 | 15 | 13 | 12 | 10 | 7 |
| **average membership probability** | 0.979 | 0.997 | 0.978 | 0.982 | 0.984 | 0.94 | 0.992 | 0.987 | 0.932 |

Repartition of the Moroccan populations from the different regions among the 9 clusters identified by DAPC, and quality measures of the assignments to clusters
